# Prognostic Impact of AJCC/UICC 8th Edition New Staging Rules in Oropharyngeal Squamous Cell Carcinoma

**DOI:** 10.3389/fonc.2017.00129

**Published:** 2017-06-30

**Authors:** Nora Würdemann, Steffen Wagner, Shachi Jenny Sharma, Elena-Sophie Prigge, Miriam Reuschenbach, Stefan Gattenlöhner, Jens Peter Klussmann, Claus Wittekindt

**Affiliations:** ^1^Department of Otorhinolaryngology, Head and Neck Surgery, University Hospital Giessen, Giessen, Germany; ^2^Department of Applied Tumor Biology, University of Heidelberg, Heidelberg, Germany; ^3^Institute of Pathology, University of Giessen, Giessen, Germany

**Keywords:** oropharyngeal cancer, surgery, human papilloma virus, prognosis, head and neck cancer, UICC 7, UICC 8

## Abstract

**Introduction:**

The purpose of this study was to test whether the 8th edition of the AJCC/UICC TNM staging system (UICC) precisely differentiates between stages and reflects disease outcome in human papilloma virus (HPV)-associated oropharyngeal squamous cell carcinoma (OPSCC).

**Patients and methods:**

OPSCC patients that were diagnosed between 2000 and 2016 were included in this analysis and HPV status was determined by combined DNA and p16 testing. Stratification was done according to 7th and 8th UICC staging rules. Incidence trends of HPV-associated tumorigenesis, 5-year overall survival (OS) according to tumor stages as well as the influence of therapy and prognostic factors toward the outcome were calculated using Kaplan–Meier method and Cox proportional-hazards model.

**Results:**

A significant increase [2000; *n* = 8/39 (21%)–2015; *n* = 17/32 (53%); *p* = 0.002] in HPV-associated OPSCC was seen in the observation period. Together, 150/599 (25.0%) of the patients had HPV-driven OPSCC and 64.7% of curative treatments in all OPSCC patients included upfront surgery of the primary and the neck. 7th edition staging rules led to no discrimination in all respective four UICC stages in HPV OPSCC underlining the need for new staging rules. However, only discrimination between stages I vs. II and III vs. IV was significant in our patients with HPV-OPSCC (94.4 vs. 77.5%; *p* = 0.031 and 63.9 vs. 25.0%; *p* = 0.013), and stages II vs. III did not differ in OS rates (*p* = 0.257), when applying the new staging rules. For HPV-negative OPSCC, significant outcome differences were only seen between UICC stages III vs. IV (57.6 vs. 35.2%; *p* = 0.012).

**Discussion:**

While the 7th edition of UICC shows invalid discrimination between stages, the 8th edition is more suitable for HPV-associated carcinoma. Due to lack of differentiation between stages II and III further adaption is essential.

## Introduction

Rising incidence rates of oropharyngeal squamous cell carcinoma (OPSCC) in several geographical areas have been reported over the last decades (1–3). A possible reason for this increase could be the sexual transmission of human papilloma virus (HPV), primarily through orogenital intercourse. After pooled analysis of 8 multinational studies with 5,642 patients with head and neck cancer and 6,069 controls, the risk of developing OPSCC was attributed with more lifetime sexual partners, more lifetime oral sex partners, and an earlier age at first sexual intercourse (4). This rising incidence is in contrast to alcohol and tobacco-related head and neck cancers, which has remained constant or is in decline (5). HPV-related OPSCCs compose a distinct entity with regard to cellular, biologic, and clinical characteristics (6, 7). Retrospective and prospective studies show that patients suffering from HPV-related OPSCC have advanced N-status compared to patients with HPV-negative OPSCC and contrary to that, significantly better local–regional control and survival after therapy (8–10).

In locally advanced disease, treatment strategies mainly consist of concomitant chemoradiotherapy (CRT) or surgery, followed by risk-adapted radio(chemo)-therapy (SRT). To date, no treatment strategy has been identified as more effective, and no predictive factors guide treatment decisions between SRT and CRT. Therefore, treatment strategies are diverse and the choice between surgical and non-surgical approaches often depend on regional preference to a large extent. However, due to favorable outcomes after CRT in HPV-associated OPSCC (8) and a lack of evidence for the benefit of surgery in OPSCC, the management with ablative surgery has been questioned (11–13).

Prognosis and treatment of solid cancers very much correlate to anatomical extent of the disease. The TNM system is generally applied for uniform description of tumor growth and spread. TNM categories lead to four stages in head and neck cancer according to published rules (14). As patients with same TNM-stages possibly show diverse survival, respective current TNM staging and stage grouping rules are constantly improved over time, particularly with respect to newly identified biomarkers. For example, minor impact of existing N-category has been shown for HPV-related OPSCC (15, 16). HPV status has emerged as the dominant prognostic biomarker in OPSCC in the last two decades. Consistently, within the latest release of AJCC cancer staging manual HPV-driven OPSCC has been described as a distinct tumor entity with different staging rules (17). The aim of this study was to test whether the latest version of TNM and UICC/AJCC stage groupings (UICC) is suitable for risk stratification in an unselected cohort of OPSCC patients with low HPV prevalence and favored SRT.

## Materials and Methods

### Patients, Tumor Samples, and Clinical Data Collection

According to International Classification of Diseases for Oncology C01, C02, C09, and C10 (ICD-O), 698 cases with OPSCC and sufficient tumor samples available diagnosed between 01/01/2000 and 07/15/2016 were identified from a tumor registry and verified by checking patients charts, and histology reports to confirm correct anatomical classification. The registration of address office was contacted for patients without documented death (key date 11/31/2016) to adjust survival data. After exclusion of 99 patients with missing HPV status (69) or unknown TNM-stage/therapy regime (30), 599 cases were identified for the current study. Written informed consent was obtained from all patients and patient data and tumor material were used in accordance and after approval by the regional ethics committee (AZ: 296/11). Tumor staging was assessed according to the UICC 7th and 8th version (clinical/pathological). All patients’ charts were reviewed for tumor characteristics (TNM), prognostic factors (performance status/ECOG, history of smoking, and alcohol), and therapy [surgery, radiotherapy (RT) and chemotherapy (CT)].

### Changes in the AJCC/UICC 8th Edition

Due to rising incidences of HPV-associated OPSCC (presented by own data), new staging rules where established for better discrimination of stages (18). As these patients with HPV-associated OPSCC usually present with better ECOG, at younger age and smaller tumors with advanced stage of regional metastasis, the AJCC/UICC 7th edition failed to differentiate properly between UICC stages. The new classification provides guidelines for p16 testing in OPSCC, in which diffuse p16 staining of ≥75% and moderate staining intensity is classified as p16 positivity and plausible HPV-related etiology. The T-category remains the same in both OPSCC entities, besides one exception for HPV-associated OPSCC: the classification does no longer contain the subdivision of T4 category into 4a and 4b. In both, HPV-associated and HPV-negative OPSCC, T0 is no longer included, but has been reassigned to a new category of cancer of unknown primary.

According to therapy options (SRT/CRT), a clinical and pathological classification has been established. Whereas T-classification remains the same in both categories (therapy) and entities, there have been major changes to N-category, especially in HPV-associated OPSCC. Clinical N-classification (cN) 1 is defined as ipsilateral lymph nodes, less than 6 cm in size, and independent of numbers. Lymph nodes less than 6 cm in size, but found ipsi- and contralateral equals cN2. cN3 presents lymph nodes bigger than 6 cm in size. Pathological N-categories (pN) are defined as pN1 correlates to lymph node metastasis ≤4 in numbers, whereas pN2 correlates to ≥5 in numbers.

N-categories for HPV-negative OPSCC have broadly remained the same, but extracapsular spread (ECS), independent of lymph node size, is now classified as N3b in clinical and pathological categories.

In clinical definition of UICC stages, stage I contains T1, T2, and N0 as well as N1. Stage II is defined as T1-, T2- with N2-status, or T3 with N0-2. Stage III contains any T- and any N-classification. Furthermore, any T- and any N-status with positive M-status is defined as stage IV and no further subdivision is done.

Pathological UICC stages are defined as following: stage I again is defined as T1, T2, and N0 as well as N1. Stage II contains T1 and T2 with N2-status or T3 with N0 or N1. Stage III is defined as T3 or T4 with N2-status and stage IV contains any T- or N-with positive M-status.

Main changes can be seen in lower UICC stages as higher T- and N-status are included compared to AJCC/UICC 7th edition and stage IV is solely defined by distant metastasis.

Definition of UICC stages in HPV-negative OPSCC remained the same.

### Therapy

Patients were diagnosed and treated following local guidelines between 2000 and 2016. HPV status was determined retrospectively when missing. Treatment was defined as the first course of OPSCC-specific treatment performed to treat the primary. Subsequent therapy to treat recurrence was not considered. Patients with OPSCC were treated with RT or surgery alone in stage I and stage II disease upon patient’s decision. Advanced cases (stage III–IVa) were allocated to surgery followed by risk-adapted SRT or CRT upon patient’s decision. Patients with unresectable disease (stage IVb) were treated without upfront surgery by means of RT or CRT depending on the performance. Patients ineligible for curative therapy (stage IVc, comorbidity, rejection of therapy within 6 weeks from date of diagnosis) received CT of best supportive care. Upfront surgery in small primary tumors (T1–2) consisted of transoral laser microsurgery. Combined approaches including pharyngotomy with composite resection of surrounding soft tissue for the primary and defect filling by free tissue transfer was performed in locally advanced tumors (T3–4). Confinement to the lateral wall only led to unilateral neck dissection. All other tumors were also treated with contralateral neck dissection. Adjuvant RT was applied with standard fractionation (1.8–2 Gy daily) to both sides of the neck area, within 6–10 weeks after surgery. Adjuvant CT was directed by pathological prognostic factors (involved surgical margins, extracapsular nodal spread, N3 status) and consisted of cisplatinum 30–40 mg/sqm weekly. CRT patients received intensity-modulated radiotherapy, combined with cisplatinum 100 mg/sqm every 3 weeks or fluorouracil 600 mg/sqm on days 1–5 plus mitomycin C 10 mg/sqm on days 5 and 36.

### Histopathology, HPV-DNA Genotyping, and p16INK4a Immunohistochemistry

Histological grading was performed following the WHO criteria for squamous cell carcinomas of the oral mucosa (19). Formalin-fixed, paraffin-embedded (FFPE) samples containing pre-therapeutic tumor tissue from patients with confirmed diagnosis were ascertained in participation with pathologists for the analysis. Tumors were classified HPV associated only when a combination of HPV-DNA and p16INK4 positive were found. For detection, DNA was extracted from variable numbers of FFPE tissue sections depending on the tissue size (10 µm sections, approximately corresponding to 10 mm × 10 mm tumor tissue) using the DNeasy Blood & Tissue Kit by Qiagen, Hilden, Germany, according to the manufacturer’s instructions. Extracted DNA was analyzed for mucosal high-risk HPV DNA and HPV genotypes (16, 18, 31, 33, 35, 39, 45, 51, 52, 56, 58, 59, 68, 73, and 82) by PCR optimized for DNA extracted from FFPE tissue, followed by bead-based hybridization (Luminex Technology, Multimetrix, Progen, Heidelberg), as described previously (20, 21). HPV-DNA contamination protection steps (e.g., new microtome blades for every block, separated pre- and post-PCR areas) were applied and appropriate controls (tissue-free paraffin blocks, water controls) were processed to monitor potential HR–HPV DNA contamination. Amplification of beta-globin was used to ensure DNA integrity. In case of no beta-globin amplification, DNA extraction was repeated from additional tumor sections to increase DNA yield. FFPE tissue sections were stained according to antibody suppliers and standard protocols. In brief, p16INK4a expression was detected using the CINtec Histology kit (Roche mtm Laboratories, Mannheim, Germany).

### Statistical and Survival Analysis

Statistical analyses were performed using SPSS statistical software (IBM Corp., Released 2016. IBM SPSS Statistics for Windows, Version 24.0.: IBM Corp., Armonk, NY, USA). Overall survival (OS) was calculated from initial date of histological diagnosis by routine biopsy to date of death. Follow-up time of event-free patients was not censored. A linear regression model was performed to conduct trend analysis for HPV association. Trend analysis was conducted for 8-year period groups over the entire period from 2000 to 2007 and 2008 to 2015. OS rates were calculated by the Kaplan–Meier method, and the significance of differences was calculated by log-rank test. Qualitative and quantitative data were compared using Pearson chi-square test. Cox proportional-hazards models were used to estimate hazard ratios (HR) with a confidence interval (CI) of 95% for OS in univariate and multivariate analysis. *p* Values ≤0.05 were considered significant for all tests.

## Results

### Biometric Data in OPSCC Patients

According to 599 patients with OPSCC the median age was 59.7 years (range 36.9–91.8), 466 (77.8%) patients were male, 385 had a good performance status (ECOG 0–1; 64.3%), 121 (20.2%) were never smokers, and 288 (48.1%) had less than 2 standard drinks per day.

### Prevalence of HPV According to Prognostic Factors and Biometric Data

**Figure 1:**
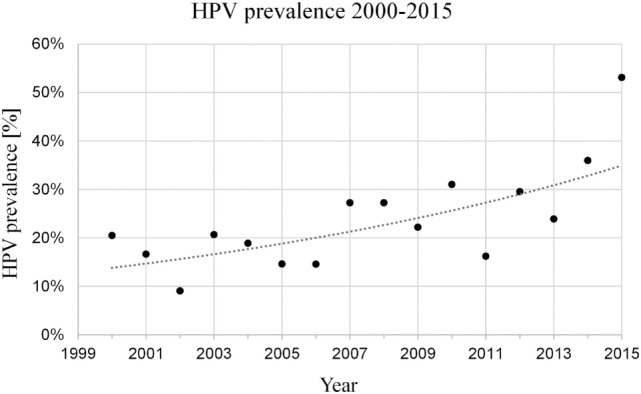
Significant increase in prevalence of human papilloma virus (HPV)-association (21–53%) in oropharyngeal squamous cell carcinoma diagnosed between 2000 and 2015 [*p* = 0.002 (Pearson), rho = 0.695 (Spearman)].

Human papilloma virus diagnostics revealed 150/599 (25.0%) patients with HPV-associated carcinoma (Table 1). An increasing amount of cases with a significant change of HPV relation in OPSCC (*p* < 0.002) could be seen in this 16-year period, mostly during 8th year period from 2008 to 2015 (Figure 1). HPV status was further assessed according to biometric data and patient possible prognostic factors for OPSCC (Table 1). HPV association was independent of age, performance, and gender (*p* = 0.556, 0.373, and 0.129). Significantly lower rates of HPV association were seen in patients with reported alcohol and nicotine abuse (*p* < 0.001). Distribution of stages determined according to both, 7th and 8th edition of UICC, showed, respectively, significant differences in the HPV associated compared to HPV-negative group (*p* < 0.001), with a significant shift toward UICC stages I–II in HPV-associated disease.

**Table 1:** Characteristics of 599 patients with oropharyngeal cancers and known human papilloma virus (HPV) status.

Characteristics of all patients—no. (%)		HPV associated 150 (25.0%)	HPV negative 449 (75.0%)	*p*-Value*
Gender	Female—133 (22.2)	40 (30.1)	93 (69.9)	0.129
	Male—466 (77.8)	110 (23.6)	356 (76.4)	

Age	Young (<60 years)—300 (50.1)	72 (24)	228 (76)	0.556
	Old (≥60 years)—299 (49.9)	78 (26.1)	221 (73.9)	

ECOG	Moderate [0–1]—385 (64.3)	91 (23.6)	294 (76.4)	0.373
	Severe [2–4]—159 (26.5)	32 (20.1)	127 (79.9)
	Unknown—55 (9.2)	27 (49.1)	28 (50.9)

Alcohol	No (<2 standard drinks)—288 (48.1)	118 (41)	170 (59)	**<0.001**
	Yes (≥2 standard drinks)—288 (48.1)	24 (8.3)	264 (91.7)	
	Unknown—23 (3.8)	8 (34.8)	15 (65.2)

Smoking	No—121 (20.2)	71 (58.7)	50 (41.3)	**<0.001**
	Yes—461 (77)	73 (15.8)	388 (84.2)	
	Unknown—17 (2.8)	6 (35.3)	11 (64.7)	

Tumor stage, 7th edition	I—62 (10.4)	4 (6.5)	58 (93.5)	**<0.001**
	II—52 (8.7)	6 (11.5)	46 (88.5)	
	III—99 (16.5)	37 (37.4)	62 (62.6)	
	Any IV—386 (64.4)	103 (26.7)	283 (73.3)	

Tumor stage, 8th edition	I—137 (22.9)	79 (57.7)	58 (42.3)	**<0.001**
	II—77 (12.9)	31 (40.3)	46 (59.7)	
	III—93 (15.5)	31 (33.3)	62 (66.7)	
	Any IV—292 (48.7)	9 (3.1)	283 (96.9)	

Therapy	Curative therapy—561 (93.7)	144 (25.7)	417 (84.3)	0.174
	Non-curative therapy (BSC)—38 (6.3)	6 (15.8)	32 (84.2)	

*p-Values calculated by Pearson’s chi-square-test (χ^2^), bold: significant values ≤ 0.050.

### Prognostic Factors According to Therapy

38 patients not suitable to curative therapy received best supportive care or palliative therapy and were excluded when further analyzing possible prognostic factors of the patients. There were 363 patients were treated with upfront surgery, while 198 patients received RT or CRT without upfront surgery (Table 2). Distribution of therapy did not differ according to HPV results (*p* = 0.168). However, patients with younger age (*p* = 0.014) and favorable performance ECOG (*p* < 0.001) were more often treated with upfront surgery. Distribution of stages determined according to 7th and 8th edition of UICC showed significant differences in the group treated with SRT compared to patients with primary RCT (*p* < 0.001, Table 2).

**Table 2:** Prognostic factors in 561 consecutive patients with oropharyngeal cancer before curative therapy.

Prognostic factors		Upfront surgery—no. (%) 363 (64.7)	No upfront surgery—no. (%) 198 (35.3)	*p*-Value*
Human papilloma virus (HPV)-association	Yes	100 (69.4)	44 (30.6)	0.168
	No	263 (63.1)	154 (36.9)	

Gender	Female	86 (68.3)	40 (31.7)	0.344
	Male	277 (63.7)	158 (36.3)	
Age	Young (<60 years)	197 (69.6)	86 (30.4)	**0.014**
	Old (≥60 years)	166 (59.7)	112 (40.3)	

ECOG	Moderate [0–1]	268 (71.5)	107 (28.5)	**<0.001**
	Severe [2–4]	60 (42.9)	80 (57.1)	
	Unknown	35 (76.1)	11 (23.9)	

Alcohol	No (<2 standard drinks)	189 (68.5)	87 (31.5)	0.083
	Yes (≥2 standard drinks)	162 (61.4)	102 (38.6)	
	Unknown	12 (57.1)	9 (42.9)	

Smoking	No	82 (71.3)	33 (28.7)	0.093
	Yes	271 (62.9)	160 (37.1)	
	Unknown	10 (66.6)	5 (33.4)	

Tumor stage, 7th edition	I	61 (100)	0	**<0.001**
	II	50 (96.2)	2 (3.8)	
	III	82 (82.8)	17 (17.2)	
	Any IV	170 (48.7)	179 (51.3)	

Tumor stage, 8th edition	I	57 (100)	0	**<0.001**
	II	45 (97.8)	1 (2.2)	
HPV-negative	III	49 (79)	13 (21)	
	Any IV	112 (44.4)	140 (55.6)	

Tumor stage, 8th edition	I	72 (92.3)	6 (7.7)	**<0.001**
	II	17 (54.8)	14 (45.2)	
HPV-positive	III	10 (34.5)	19 (65.5)	
	Any IV	1 (16.7)	5 (83.3)	

*p-Values calculated by Pearson’s chi-square-test (χ^2^), bold: significant values ≤ 0.050.

### Univariate Overall Survival Analysis According to Prognostic Factors, Tumor Characteristics, and Treatment

The 5-year OS of the study group was 54.5% (HPV related: 82.8% vs. HPV-negative: 45.5%). In median survival analysis, patients with younger age (<60 years), low ECOG value (0–1) and alcoholic consumption ≤2 standard drinks per day had significantly better OS rates (*p* < 0.001, Table 3). Furthermore, no history of smoking (*p* = 0.002) also resulted in better OS. Patients who were treated with upfront surgery also had significantly longer OS, including all stages and after excluding advanced T-status for BIAS correction (<0.001). When separately comparing UICC stages I–IV 7th vs. 8th edition, stage groupings I (trend) and IV result in significant changes toward the outcome, while stage grouping II and III do not (Table 3). This change of stage I toward a better outcome and stage IV toward an unfavorable outcome leads to a better separation of respective survival curves when comparing both models.

**Table 3:** Prognostic factors for survival in patients with oropharyngeal cancer after curative therapy and with known human papilloma virus (HPV) status.

Factor	Group	No.	Survival (univariate)				Hazard ratios (HR) (95% CI)	*p*-Value**	Multivariate analyses	
			Median overall survival (OS) (years)	2-year OS (%)	5-year OS (%)	*p*-Value*			HR (95% CI)	*p*-Value***
All		561	5.723	71.7	54.5	–				

HPV	Yes	144	–	88	82.8	**<0.001**	0.316	**<0.001**		
	No	417	7.414	66.4	45.5					

Gender	Female	126	7.551	73.3	59.3	0.397	1.140	0.396	1.033	0.865
	Male	435	5.537	71.2	53					

Age	Young (<60 years)	283	8.838	77.7	61.7	**<0.001**	1.627	**<0.001**	1.460	**0.018**
	Old (≥60 years)	278	4.625	65.5	46.5					

ECOG	Moderate [0–1]	375	7.633	79.1	62.2	**<0.001**	2.507	**<0.001**	1.644	**0.004**
	Severe [2–4]	140	1.915	48.3	30.6					

Alcohol	No (<2 standard drinks)	276	–	78.9	66.1	**<0.001**	2.059	**<0.001**	2.357	**<0.001**
	Yes (≥2 standard drinks)	264	4.480	66.2	45.5					

Smoking	No	115	–	78.9	66.8	**0.002**	1.801	**0.002**	1.253	0.355
	Yes	431	5.293	66.2	45.5					

Therapy	Upfront surgery	363	11.178	84.8	68.4	**<0.001**	0.332	**<0.001**		
	No upfront surgery	198	1.858	47.4	28.8					

Therapy (T1–T3)	Upfront surgery	339	11.178	85.2	68.5	**<0.001**	0.400	**<0.001**	0.461	**<0.001**
	No upfront surgery	49	2.405	56.1	38.6					

UICC stage I	7th ed.	62	12.274	88.2	61.2	0.062	0.880	0.066		
	8th ed.	137	–	91.2	97					

UICC stage II	7th ed.	52	11.321	94.2	70.7	0.112	1.027	0.740		
	8th ed.	77	11.321	93.2	69.5					

UICC stage III	7th ed.	99	7.200	80.4	70.7	0.296	1.061	0.299		
	8th ed.	93	6.216	72	59.5					

UICC stage IV	7th ed.	386	3.063	58.7	43	**0.007**	1.070	**0.008**		
	8th ed.	292	2.011	50.4	32.5					

*p-Value calculated by log-rank (Kaplan–Meier) test.

**p-Value calculated by log-rank (Mantel–Cox) test; univariate.

***p-Value calculated by log-rank (Mantel–Cox) test; multivariate, bold: significant values ≤ 0.050.

### Univariate Overalls Survival Analysis According to Prognostic Factors, Tumor Characteristics, and Treatment in Patients with HPV-Associated Tumors

**Figure 2:**
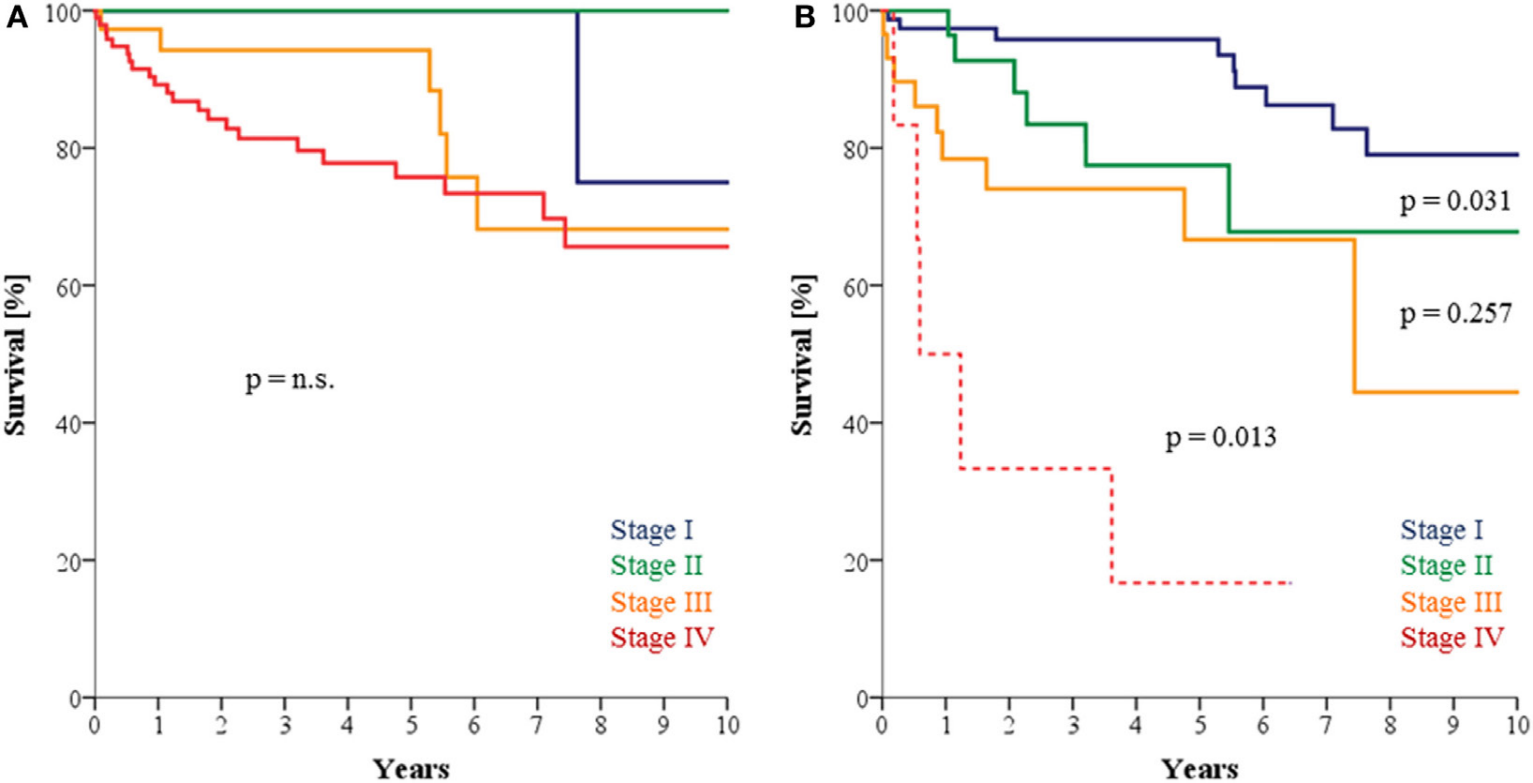
**(A)** Survival of patients with human papilloma virus (HPV)-associated oropharyngeal squamous cell carcinoma (OPSCC) according to UICC 7th edition which presents with poor discriminative power between stages. **(B)** Survival of patients with HPV-associated OPSCC according to UICC 8th edition with significant discriminative power between stages I vs. II and III vs. IV but not between stages II vs. III.

After analysis of the prognostic factors in oropharyngeal cancer patients with HPV-associated tumors, a significantly better OS can be seen in younger patients (<60 years) and patients with favorable performance (ECOG 0–1). Upfront surgery also resulted in longer 5 years OS in OPSCC patients with HPV-related tumors (all patients: SRT 93% vs. CRT 57.7%, *p* < 0.001; T4 excluded: SRT 93.4% vs. CRT 73.4%, *p* = 0.009). When comparing UICC stages 7th vs. 8th edition, a significant deterioration of 5 year OS in stage groups III (trend) and IV became apparent. The survival rates in stages I and II showed no significant difference in compliance with low patient numbers in UICC 7th edition stage groups I and II (Table 4). The survival curves in HPV-related OPSCC accordingly depicted better discriminative power between the subgroups after applying 8th edition changes (Figure 2). Smoking and alcohol had no significant influence on OS in patients with HPV-associated OPSCC.

**Table 4:** Prognostic factors for survival in human papilloma virus-associated cancer of the oropharynx.

Factor	Group	No.	Survival (univariate)				Hazard ratios (HR) (95% CI)	*p*-Value**	Multivariate analyses	
			Median overall survival (OS) (years)	2-year OS (%)	5-year OS (%)	*p*-Value*			HR (95% CI)	*p*-Value***
Gender	Female	39	–	89.4	80.9	0.690	1.188	0.690		
	Male	105	–	87.5	83.3					

Age	Young (<60 years)	69	–	93.7	91.7	**0.003**	3.247	**0.005**	2.342	0.094
	Old (≥60 years)	75	11.468	82.9	74.3					

ECOG	Moderate [0–1]	90	–	93.2	91.7	**<0.001**	3.545	**0.001**	0.695	0.695
	Severe [2–4]	31	7.438	70.3	55.3					

Alcohol	No (<2 standard drinks)	113	–	87.6	83.9	0.111	1.948	0.117		
	Yes (≥2 standard drinks)	23	7.438	90.6	82.4					

Smoking	No	69	–	87.5	83.3	0.236	1.592	0.240		
	Yes	69	11.468	87.7	83.1					

Therapy	Upfront surgery	100	–	97	93	**<0.001**	0.206	**<0.001**		
	No upfront surgery	44	7.438	65.8	57.7					

Therapy (T1–T3)	Upfront surgery	92	–	97.8	93.4	**0.009**	0.300	**0.013**	0.394	0.071
	No upfront surgery	24	7.438	79.5	73.4					

UICC stage I	7th ed.	4	–	75.0	75.0	0.968	1.011	0.968		
	8th ed.	79	–	94.4	94.4					

UICC stage II	7th ed.	6	–	–	–	0.138	2.429	0.374		
	8th ed.	31	–	92.7	77.5					

UICC stage III	7th ed.	37	11.468	97.3	94.3	0.055	1.271	0.064		
	8th ed.	31	7.438	71.0	63.9					

UICC stage IV	7th ed.	103	–	82.5	74.2	**0.003**	1.407	**0.006**		
	8th ed.	9	1.233	50.0	25.0					

*p-Value calculated by log-rank (Kaplan–Meier) test.

**p-Value calculated by log-rank (Mantel–Cox) test; univariate.

***p-Value calculated by log-rank (Mantel–Cox) test; multivariate, bold: significant values ≤ 0.050.

### Univariate Overall Survival Analyses According to Prognostic Factors, Tumor Characteristics, and Treatment in Patients with HPV-Negative Tumors

Patients with an HPV-negative tumor, younger age (<60 years, *p* = 0.001), low ECOG (stage 0–1, *p* < 0.001), and alcohol consumption ≤2 standard drinks per day (*p* = 0.003) resulted in longer OS. The 5-year-OS rates significantly differ between treatments (SRT: 59.5% vs. CRT: 22%; HR 0.364, *p* < 0.001). After adjusting for moderate T-status, the survival benefit between both groups was still present (SRT 60.4% vs. CRT 28.5%, HR 0.410, *p* < 0.001; Table 5).

**Table 5:** Prognostic factors for survival in human papilloma virus-negative patients with oropharyngeal cancer.

Factor	Group	No.	Survival (univariate)				Hazard ratios (HR) (95% CI)	*p*-Value**	Multivariate analyses	
			Median overall survival (OS) (years)	2-year OS (%)	5-year OS (%)	*p*-Value*			HR (95% CI)	*p*-Value***
Gender	Female	87	5.197	66.6	50.6	0.711	1.063	0.711	1.005	0.979
	Male	330	4.364	66.4	44					

Age	Young (<60 years)	214	5.538	73.1	52.9	**<0.001**	1.582	**0.001**	1.417	**0.043**
	Old (≥60 years)	203	2.866	59.1	37.1					

ECOG	Moderate [0–1]	285	5.323	74.7	53.1	**<0.001**	2.480	**<0.001**	1.904	**<0.001**
	Severe [2–4]	109	1.668	41.9	23.3					

Alcohol	No (<2 standard drinks)	163	5.641	73	53.9	**0.003**	1.540	**0.003**	1.872	**0.001**
	Yes (≥2 standard drinks)	241	3.874	64	42.5					

Smoking	No	46	4.616	72.8	40.8	0.879	1.035	0.879	0.800	0.427
	Yes	362	4.408	66.3	46.5					

Therapy	Upfront surgery	263	6.655	80.3	59.5	**<0.001**	0.364	**<0.001**		
	No upfront surgery	154	1.718	42.7	22					

Therapy (T1–T3)	Upfront surgery	247	6.655	80.7	60.4	**<0.001**	0.410	**<0.001**	0.483	**<0.001**
	No upfront surgery	70	1.929	49	28.5					

*p-Value calculated by log-rank (Kaplan–Meier) test.

**p-Value calculated by log-rank (Mantel–Cox) test; univariate.

***p-Value calculated by log-rank (Mantel–Cox) test; multivariate, bold: significant values ≤ 0.050.

### Multivariate Analysis of Overall Survival

To further describe BIAS after summation of possible prognostic factors in the SRT group leading to superior OS a multivariate analysis was performed. Gender, age, performance, alcohol, history of smoking and therapy (T1–T3) were included in the analysis. The Cox multivariate analysis suggested that besides age, ECOG, and alcohol therapy (T1–T3, HR 0.461; *p* < 0.001) is an independent predictor for better OS in all OPSCC patients (Table 3). When analyzing age, ECOG, and therapy (T1–T3) according to HPV, the Cox multivariate analysis was not able to confirm any of the abovementioned possible prognostic factors, though a trend for age and therapy could be seen (Table 4). In OPSCC patients with HPV-negative tumors, however, age, ECOG, alcohol, and SRT after excluding advanced T-status were (T1–T3, HR 0.483; *p* < 0.001) presented as independent prognostic factors for a longer OS (Table 5).

### Discriminative Power of Stage Groupings in OPSCC

To further analyze and describe the discrimination of OS values in the respective stage groups, OS values and log-rank test results were displayed (Table 6). Surprisingly, for patients with HPV-negative OPSCC only for stages III/IV (*p* = 0.012; but not for stages I/II, II/III) significant discrimination between relevant adjacent stages in UICC 7th/8th edition could be depicted. Stages I and II showed similar results. Stages II/III showed nearly significant deterioration of survival. For adjacent stage groups in HPV-related disease (I/II, II/III, III/IV) in UICC 7th and 8th edition in patients with HPV-associated carcinoma, only stage I vs. stage II (*p* = 0.031) and stage III vs. IV (*p* = 0.013) showed significant discrimination for survival.

**Table 6:** Survival differences and discriminative power of stage groupings in oropharyngeal cancer.

Human papilloma virus (HPV)-associated cancer of the oropharynx [2-year OS/5-year OS]					
UICC stages	I (95.8/95.8)	II (92.7/77.5)	III (74.1/66.6)	IV (33.3/16.7)	8th edition
I		**0.031**	**0.001**	**<0.001**	I
II	0.480		0.257	**0.001**	II
III	0.388	0.139		**0.013**	III
Any IV	0.558	0.152	0.534		Any IV
7th edition	I (75.0/75.0)	II (0/0)	III (94.3/94.3)	Any IV (84.2/75.8)	

**HPV-negative cancer of the oropharynx (2-year OS/5-year OS)**					
UICC stages	I (88.9/58.1)	II (93.4/65.9)	III (72.4/57.6)	IV (54.4/35.2)	7/8th edition
		0.685	0.156	**<0.001**	I
			0.096	**<0.001**	II
				**0.012**	III
					Any IV
	I	II	III	Any IV	

*p-Value calculated by log-rank (Kaplan–Meier) test, bold: significant values ≤ 0.050; light grey: UICC 8th edition; dark grey: UICC 7th edition; white: UICC 7th/8th edition*.

## Discussion

Mean survival of OPSCC has improved drastically in recent decades, not by tremendous treatment progress, but likely due to steadily increasing rates of HPV-associated OPSCC as shown in our cohort and by others (22, 23). Although the purpose of staging systems is to provide solid information about prognosis, HPV as an essential prognostic factor was not included in 7th version of UICC. Consistently, in several publications a weak power of differentiation of UICC 7th edition in HPV-associated carcinoma has been reported, whereas acceptable results were described for HPV-negative carcinoma (15, 24, 25). The latest edition (8th UICC) considers HPV status for better differentiation between stages and prediction of survival in OPSCC based on cohorts primarily treated with CRT (25) or SRT (26). We evaluated the benefit of new staging rules in HPV-associated OPSCC in an unselected cohort of patients, predominantly treated with upfront surgery followed by adjuvant risk-adapted radio(chemo)therapy (SRT) for tumor control. Our results show significant stratification between stages I vs. II but not II vs. III in patients with HPV-associated carcinoma. This lack of stratification between stages II and III could be influenced by low number of cases and events (death) due to improved survival rates in patients with HPV-associated OPSCC. Additionally, different criteria for UICC stages in surgically and CRT-treated patients exist according to treatment modality.

The TNM criteria have been revised in the 8th edition of UICC according to a large patient cohort (*n* = 1.907 patients with HPV-associated carcinoma) mainly treated with CRT (International Collaboration on Oropharyngeal cancer Network for Staging—ICON-S). UICC stages were adapted according to similar survival rates in 7th edition (no subdivision in T4, re-termination of N-status) and new classification-terms presented with a significant differentiation (*p* < 0.001) between stages (I vs. II, II vs. III) in both cohorts (25). However, the discrimination between stages I vs. II seemed to be very low (OS rates: 85 vs. 78%) in the cohort described by O’Sullivan and coauthors. Additionally, only 1% in their respective training cohort and 2% in the validation cohort were treated with upfront surgery. Therefore, outcome measurements may not be comparable. Significantly worse outcome in stage I patients by clinical staging of lymph node involvement in CRT patients might also be explained by false negative results of clinical lymph node assessment in comparison to lymph node staging after upfront neck dissection. Finally, the authors implemented HPV association as p16INK4a-stain or *in situ* hybridization for HPV-DNA only in the ICON-S cohort. It is well known that assigning patients to HPV-related OPSCC according one-test-only harbors the risk of bias (27, 28).

To improve staging rules for patients treated with SRT, a sample of 704 patients with HPV-associated carcinoma was evaluated. When using clinical staging system on a primarily surgically treated cohort overlap of OS between stages was seen (26). Therefore, classification of N-status was adapted according to number of infiltrated lymph nodes, whereas ECS had no prognostic influence in OS in patients with HPV-associated carcinoma (29). When using those new pathological staging rules, a significant differentiation (*p* < 0.001) between stages I vs. II and II vs. III (5-year OS: I 90%, II 84%, III 48%) were seen (26). This again, stands in contrast with our results, as we found poor differentiation between stages II and III. Repeatedly, the low number of patients with HPV-associated carcinoma in our cohort and solely p16INK4a testing in this 704 patient cohort could be a reason for diverse outcome.

In a recent publication, the UICC 8th edition has been tested on 195 patients with OPSCC (111 HPV-associated, confirmed only by p16INK4a-status) treated with SRT or CRT in Japan (30). UICC stages I and II were condensed due to similar survival rates (3-year OS: I 90.9, II 90.9, III 70.2) and presented with significant differentiation compared to stage III (*p* < 0.001) in this retrospective study. This again stands in contrast to our results and might be due to summarization of stages, inaccurate HPV testing, and improper classification of N-category. Although patients were treated with SRT and CRT, it is reported that classification was performed according to clinical stages only.

As evaluation in UICC 8th edition is done separately according to different criteria for either patients treated with SRT or CRT, choice of therapy plays a pivotal role. Since no therapy of choice has been identified in OPSCC independent of HPV status, to this point no evidence for better outcome or better classification has been reported. Due to favorable outcome after therapy in HPV-associated OPSCC treatment, modality seems to be of minor significance for the outcome, whereas in HPV-negative tumors, upfront surgery might be an independent factor for improved OS (own unpublished data). Although in this retrospective study, the Cox multivariate analysis identified age, ECOG, alcohol and therapy as an independent predictor for better OS in all OPSCC, results have to be interpreted with caution due to an unavoidable selection bias in patients. Further studies are needed to investigate the role of treatment modality in OPSCC as this has an impact on discrimination of stages in UICC classification and furthermore, proper classification plays a pivotal role for eventual consideration of de-escalation of treatment in HPV-associated OPSCC.

Inclusion of further possible prognostic factors such as smoking, age, performance status, quality of life, EGFR-expression, p53 status, Bcl-2, and ERCC1, as recommended by UICC (18), could improve stratification in OPSCC. In multiple cohorts, smoking and TNM status were reported to effect OS (8, 25, 29, 31), which is in contrast to our findings as we found alcohol, but not smoking, had a significant impact on the outcome of OPSCC patients. In our model, smoking might be canceled out due to the high rate of smokers in the HPV-negative group. Furthermore, smoking might influence radiosensitivity, e.g., *via* reducing tumor oxygenation by a rise in carboxyhemoglobin level in smokers and its prognostic impact could be less important after primary surgical treatment. In further studies by our group, T-status in patients with HPV-associated OPSCC, performance status, N-category, and age in patients with HPV-negative OPSCC were identified in a RPA model to be important predictors for prolonged OS after SRT (32), whereas we found that the model, introduced by Ang et al., seems to be unsuited for unselected, primary surgically treated patients (8).

There are certain drawbacks in our study: a single institution cohort, limitations of retrospective data evaluation, particularly on tobacco and alcohol consumption. Additionally, classification of HPV-negative OPSCC was done according to 7th edition of UICC as this was the applied staging system in the time period of treatment in the described cohort. Therefore, ECS has not been documented on a regular basis. As this important factor has been introduced in 8th edition (5-year OS: N0 85.5%, N+/ECS− 62.5%, N+/ECS+ 29.9%) (33, 34) upcoming analysis has to include ECS for validation of proper discrimination in new AJCC stage groupings.

## Conclusion

Our data demonstrate that stratification according to HPV, and changes in TNM categories in the 8th edition of AJCC/UICC improve discriminative power in a non-selected cohort of OPSCC patients primarily treated with upfront surgery. However, distinction between stages II and III in patients with HPV-associated OPSCC seems to be poor. Additionally, we confirmed alcohol to be an important prognostic factor in HPV-negative OPSCC, whereas smoking and alcohol had no significant influence on OS in patients with HPV-associated OPSCC. In conclusion, the 8th edition of AJCC/UICC TNM staging system is more suitable for HPV-associated carcinoma although further adaptation may be needed to improve prediction of prognosis.

## Ethics Statement

The protocol was approved by the “regional ethics committee.” Written informed consent, in accordance with the Declaration of Helsinki, was obtained from patients to use archived samples for scientific purpose.

## Author Contributions

Each author has provided substantial contributions to warrant authorship. Specific contributions are as follows. NW and SW: conceptualization, resources, data curation, formal analysis, investigation, methodology, project administration, supervision, validation, visualization, writing-original draft, writing-review, and editing. SS: resources, data curation, validation, writing-review, and editing. MR, E-SP, and SG: conceptualization, resources, formal analysis, validation, writing-review, and editing. JK and CW: conceptualization, resources, investigation, methodology, supervision, visualization, writing-original draft, writing-review, and editing.

## Conflict of Interest Statement

The research was conducted in the absence of any commercial or financial relationships that could be construed as a potential conflict of interest. No conflict of interest to disclose.
